# Predicting In-Hospital Mortality in Acute Mesenteric Ischemia: The RADIAL Score

**DOI:** 10.3390/jcm15031106

**Published:** 2026-01-30

**Authors:** Luis Castilla-Guerra, Paula Luque-Linero, Maria del Carmen Fernandez-Moreno, Belén Gutiérrez-Gutiérrez, Francisco Fuentes-Jiménez, María Adoración Martín-Gómez, María Dolores Martínez-Esteban, María del Pilar Segura-Torres, Maria Dolores López-Carmona, Patricia Rubio-Marín

**Affiliations:** 1 Vascular Risk Unit, Internal Medicine Department, Hospital University Virgen Macarena, 41009 Seville, Spain; castillafernandez@hotmail.com; 2 Department of Medicine, University of Seville, 41009 Seville, Spain; cafemo101@us.es (M.d.C.F.-M.); bgutierrez2@us.es (B.G.-G.); 3 Neurology Department, Virgen de Valme Hospital, 41014 Seville, Spain; 4 Clinical Unit of Infectious Diseases and Microbiology, Hospital University Virgen Macarena, 41009 Seville, Spain; 5 Internal Medicine Department, Reina Sofía University Hospital, 14004 Córdoba, Spain; francisco.fuentes@upm.es; 6 Faculty of Medicine, University of Medicine of Córdoba, 14071 Córdoba, Spain; 7 Nephrology Department, Poniente University Hospital, 04700 El Ejido, Spain; doritamg@gmail.com; 8 Nephrology Department, Regional University Hospital, 29010 Málaga, Spain; lolamares1@gmail.com; 9 Nephrology Department, University Hospital of Jaen, 23007 Jaén, Spain; psegurat@senefro.org; 10 Internal Medicine Department, Regional University Hospital, 29007 Málaga, Spain; mdlcorreo@gmail.com; 11 Internal Medicine Department, Hospital Universitario de Jerez, 11407 Jerez de la Frontera, Spain; prubiomarin@gmail.com

**Keywords:** acute mesenteric ischemia, prognostic SCORE, mortality, risk stratification

## Abstract

**Background/Objectives:** Acute mesenteric ischemia (AMI) is a time-dependent condition associated with exceptionally high in-hospital mortality, particularly among elderly and comorbid patients. Early identification of patients at high risk of death remains challenging and has important implications for clinical decision-making. The objective of this study was to derive and internally validate a prognostic score for in-hospital mortality of patients with AMI. **Materials and Methods:** We conducted a multicenter, observational, retrospective cohort study including patients with AMI from 10 participating hospitals. A descriptive and analytical approach was performed. A Classification and Regression Tree (CART) model was used to determine cut-off points for continuous variables and assess their association with mortality. Based on these thresholds, a univariate analysis was performed, and variables with statistical significance (*p* < 0.05) were incorporated into a multivariate logistic regression model. A score—the RADIAL score—was then derived from the beta coefficients. The discriminative ability of the score was evaluated using the receiver operating characteristic (ROC) curve. **Results**: A total of 693 patients were studied. Thee mean age was 81 years (IQR 73–86) and 54.2% were women. A history of cardiovascular disease was present in 75.3% of participants. Overall mortality was 62.4%. Most patients (74%) were managed conservatively. Significant variables in the bivariate analysis included hypotension, age > 65 years, pH < 7.3, creatinine > 1.7 mg/dL, and absence of rectal bleeding. These variables were incorporated into the multivariate model. The resulting score showed an area under the ROC curve of 0.78 (95% CI: 0.74–0.82). **Conclusions**: The RADIAL score demonstrated robust predictive performance and allowed the identification of three mortality-risk groups: 30–40% (low), 50–60% (intermediate), and 80% (high). This tool may support clinical decision-making in the management of patients with AMI.

## 1. Introduction

Mesenteric ischemia results from inadequate intestinal perfusion, leading to progressive bowel injury, transmural necrosis, and potentially fatal organ failure without timely treatment [1]. This reduction in perfusion may result from arterial occlusion, venous thrombosis, or non-occlusive mechanisms related to systemic hypoperfusion, all of which share a final common pathway of intestinal hypoxia and loss of mucosal integrity. Once this cascade is initiated, bacterial translocation, systemic inflammatory response, and multiple organ dysfunction may rapidly ensue.

Although traditionally considered a low-prevalence entity, this perception is likely misleading and largely attributable to the considerable diagnostic challenges inherent to the disease. Acute mesenteric ischemia (AMI) often presents with vague and nonspecific symptoms, which overlap with a wide range of more common abdominal conditions. Laboratory tests lack both sensitivity and specificity in the early stages, and no single biomarker has demonstrated sufficient accuracy to reliably confirm or exclude the diagnosis [2,3]. As a result, diagnosis frequently relies on a high index of clinical suspicion rather than on early objective findings, contributing to substantial diagnostic delays [4,5].

As with other ischemic syndromes, time is a critical determinant of outcome in AMI [6]. Even short delays in recognition and treatment can result in irreversible intestinal injury, dramatically worsening prognosis. Once transmural necrosis develops, mortality rates increase sharply, making AMI one of the deadliest causes of acute abdomen. This is particularly evident in older adults, in whom mortality remains exceedingly high despite advances in imaging, endovascular techniques, and perioperative care [7]. The time-dependent nature of intestinal ischemia underscores the importance of early decision-making, yet this is precisely the phase in which clinical uncertainty is greatest.

Management strategies for AMI range from conservative treatment and hemodynamic optimization to endovascular revascularization and open surgical intervention, often combined with bowel resection [8]. However, AMI predominantly affects elderly patients with multiple comorbidities, including cardiovascular disease, renal dysfunction, and frailty [9]. In this population, invasive treatment does not uniformly translate into improved survival and may be associated with significant perioperative morbidity, prolonged intensive care, and poor functional recovery. Consequently, clinicians are frequently faced with the difficult task of balancing the potential benefits of aggressive intervention against the risk of offering treatments that may ultimately prove futile [10].

These challenges highlight a critical gap in current clinical practice: the lack of structured, objective, and clinically applicable tools capable of providing reliable prognostic information at the time of presentation. While several studies have identified individual clinical, laboratory, or radiological predictors of mortality in AMI, these factors are often considered in isolation and do not adequately capture the complex, multifactorial nature of the disease [11]. More accurate risk stratification could help identify patients who are likely to benefit from prompt invasive management, while sparing others from unnecessary procedures and allowing earlier consideration of conservative or palliative approaches when appropriate [10,12,13]

Therefore, the aim of this study was to develop and validate a prognostic tool designed to support clinical decision-making in patients with AMI. By integrating readily available clinical, laboratory, and radiological variables into a unified risk model, we sought to provide a practical instrument to assist clinicians in navigating the time-critical and high-stakes therapeutic decisions inherent to this condition.

## 2. Material and Methods

### 2.1. Study Design and Population

A retrospective, multicenter observational cohort study was conducted. It included all patients with AMI who were admitted to ten hospitals in Andalusia (Southern Spain) between January 2010 to December 2020.

### 2.2. Participants

Adult patients aged 18 years or older with a diagnosis of acute mesenteric ischemia were considered eligible for inclusion, provided that informed consent had been obtained. The diagnosis of acute mesenteric ischemia was established based on confirmation by at least one diagnostic modality, including computed tomography angiography, conventional arteriography, or intraoperative findings.

Patients younger than 18 years were excluded, as were those in whom mesenteric ischemia was attributable to extrinsic mechanical compression of the mesenteric vessels, such as intestinal obstruction, volvulus, compressive tumors, or abdominal compartment syndrome.

**Figure 1:**
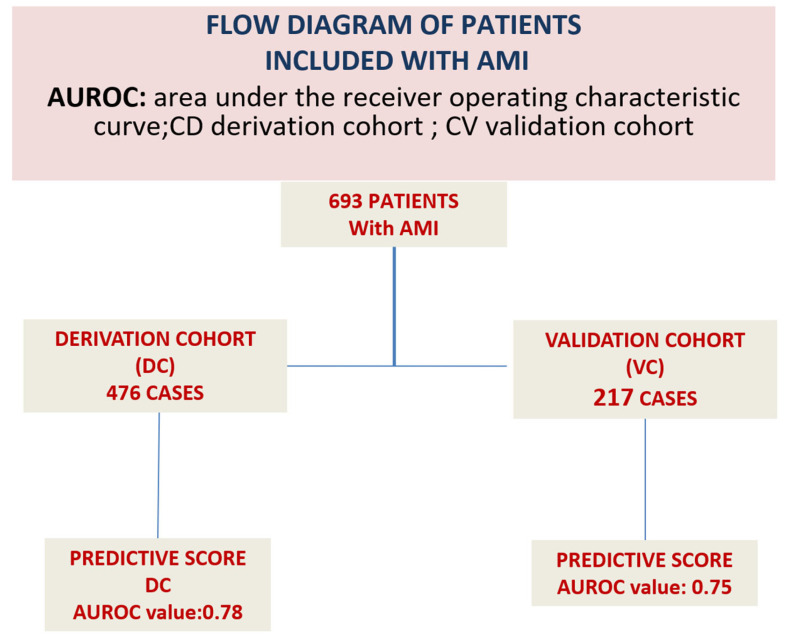
Explanation of the creation of the derivation cohort and the validation cohort for internal validation.

A total of 693 patients met the inclusion criteria and were included in the analysis. These patients were subsequently allocated into a Derivation cohort (n = 476) for development of the predictive model and a Validation cohort (n = 217) for internal validation. The study flow and cohort allocation are illustrated in Figure 1.

### 2.3. Data Collection and Clinical Assessment

Collected variables included demographic characteristics (age, sex, year of admission, hospital department of admission, baseline functional status, and length of hospital stay); cardiovascular risk factors and comorbidities (smoking status, alcohol consumption, obesity, hypertension, diabetes mellitus, dyslipidemia, chronic kidney disease, ischemic heart disease, heart failure, dementia, prior stroke or transient ischemic attack, peripheral arterial disease, previous vascular stenting, atrial fibrillation, polyvascular disease, previous malignancy, and associated neoplasia); etiology (atherosclerotic or embolic); affected artery (superior mesenteric artery, inferior mesenteric artery, or other); clinical presentation (abdominal pain, fever, vomiting, constipation, abdominal distension, rectorrhagia, hematemesis, signs of peritonitis, and arterial hypotension); diagnostic modalities (computed tomography or magnetic resonance imaging); laboratory parameters (arterial pH, C-reactive protein, lactate, glucose, lactate dehydrogenase, leukocyte count, creatine phosphokinase, urea, and creatinine); and treatment strategies and clinical outcomes (surgical management, endovascular treatment, palliative treatment, and intensive care unit admission) during the index hospitalization. For laboratory parameters, the value corresponding to the most abnormal measurement within 24 h prior to diagnosis was recorded.

### 2.4. Statistical Analysis

Descriptive analyses were performed by calculating the rates of categorical variables and the median and interquartile ranges (IQRs) for continuous variables. Categorical variables are summarized as counts and percentages. Differences between proportions were assessed using the χ^2^ test, and Student’s *t* test or the Wilcoxon rank-sum test for continuous variables were used for evaluating differences between continuous variables.

Missing data were imputed using a Markov Chain Monte Carlo method. After imputation, the dataset was randomly divided into a Derivation cohort (approximately two-thirds of the sample) and a Validation cohort (one-third of the sample). No significant baseline differences were observed between cohorts.

In the Derivation cohort, a Classification and Regression Tree (CART) model was used to identify optimal cut-off points for continuous variables and to explore potential center or temporal effects. Continuous variables were dichotomized according to these CART-derived thresholds. Univariable logistic regression was performed to identify candidate predictors; variables with *p* < 0.05 were included in a multivariable logistic regression model. Collinearity was assessed using the variance inflation factor (VIF), with values < 2 considered acceptable.

A prognostic score was constructed from the β-coefficients of the final multivariable model. The prognostic performance of the score, including sensitivity, specificity, positive predictive value (PPV), negative predictive value (NPV), and area under the receiver operating characteristic (ROC) curve, was assessed in the Derivation cohort. Internal validation was performed using the Validation cohort, confirming the robustness of the model and its ability to stratify patients into three mortality-risk groups: 30–40%, 50–60%, and ~80%.

All analyses were conducted using IBM SPSS Statistics version 28 (SPSS, Chicago, IL, USA).

### 2.5. Ethical Aspects

Ethical approval for this study was granted by the Clinical Research Ethics Committee of Hospital Universitario Virgen Macarena in compliance with Spanish Biomedical Research Law 14/2007 (approval code 1341-N-21). Written informed consent was obtained from all participants prior to participation. All procedures adhered to the approved protocol and were conducted in accordance with the Declaration of Helsinki and Organic Law 3/2018 on personal data protection and digital rights

Participants were fully informed about the study objectives and procedures. As this was a retrospective, multicenter observational study, no procedures beyond standard clinical practice were performed. The study was based exclusively on the review of patients’ clinical characteristics and available laboratory results obtained during routine care. No additional examinations, medical interventions, or tests were required, and no risks or discomfort for patients were anticipated. All clinical data were fully anonymized prior to analysis

## 3. Results

A total of 693 patients were included in the analysis. The median age was 81 years (IQR 73–86), and 54.2% were women. In-hospital mortality was high, with an overall rate of 62.4%, increasing to 70.1% among patients aged 85 years and above.

Regarding hospital admission, most patients were admitted to the Internal Medicine department (63.8%). Only 15.9% of the patients were admitted to the Surgery department.

### 3.1. Personal Background

Cardiovascular risk factors were highly prevalent in the study population, with hypertension affecting 77% of patients, followed by type 2 diabetes mellitus (43%), dyslipidemia (41%), smoking (30.7%), and obesity (21%). Overall, 74.1% of patients had established cardiovascular disease, including ischemic heart disease (28%), stroke (21%), and peripheral arterial disease (25.1%). Chronic kidney disease, atrial fibrillation, heart failure, and malignancy were among the most frequent additional comorbidities (Table 1).

### 3.2. Treatment Used

Conservative management was the predominant treatment strategy. Surgery was required in 130 patients (18.5%), and 55 patients (7.9%) received endovascular therapy.

### 3.3. Variables Related to Surgery

Baseline characteristics differed significantly between patients managed with non-surgical treatment and those who underwent surgical or endovascular intervention. Patients treated non-surgically were significantly older than those who received invasive management (mean age 79.1 ± 11 vs. 73.9 ± 11 years, *p* = 0.0001). Female sex was more prevalent in the non-surgical group (56.5% vs. 43.1%, *p* = 0.008).

Functional dependence was markedly more frequent among patients managed non-surgically (57.5% vs. 19.4%, *p* = 0.0001), as was the presence of dementia (21% vs. 1.5%, *p* = 0.0001). Comorbid conditions were also significantly more common in this group, including diabetes mellitus (47.1% vs. 32.7%, *p* = 0.003) and polyvascular disease (27.8% vs. 16.1%, *p* = 0.001).

Furthermore, a history of prior major amputation was observed exclusively in the non-surgical group (5% vs. 0%, *p* = 0.005). Overall, patients managed without surgical or endovascular intervention exhibited a greater burden of comorbidities and functional impairment, factors that may have influenced the choice of therapeutic strategy.

### 3.4. Predictors of Death

Before performing the univariate analysis, the CART model was used to establish the cut-off points of the continuous variables and their association with mortality.

Table 2 shows the cut-off of the different continuous variables. Table 3 shows the results of univariate analysis of pathology and biomarkers predictors in relation to death.

No cardiovascular risk factors showed a statistically significant relationship with mortality (*p* < 0.05). Patients who suffered from kidney disease (*p* = 0.01) and a previous stroke were related to a higher mortality (*p* = 0.013).

At admission, a diagnosis of hypotension and not having rectal bleeding were also positively related to death.

The following biomarkers were also related to worse prognosis: pH < 7.30 (*p* = 0.001), Glucose > 230 (*p* = 0.003), leukocytosis > 10,000 (*p* = 0.0002), CPK > 400 (*p* = 0.02), urea > 43 (*p* = 0.001), creatinine > 1.7 (*p* = 0.01), CRP > 50 (*p* = 0.004), LDH > 875 (*p* = 0.002). Lactic acid was not statistically significant (*p* = 0.24, *p* = 0.67).

All the identified risk factors from the univariate analysis entered a logistic regression analysis. Of the patient baseline variables, hypotension (OR 2.50 CI95% 1.65–3.79), age > 65 years (OR 2.08 CI95% 1.27–3.41), pH < 7.3 (OR 2.03 CI95% 1.38–2.98), creatinine superior > 1.7 (OR 1.60 CI95% 1.10–2.33), and not having rectal bleeding (OR 1.83 CI95% 1.76–1.89) were associated with an increased risk of in-hospital mortality.

**Figure 2:**
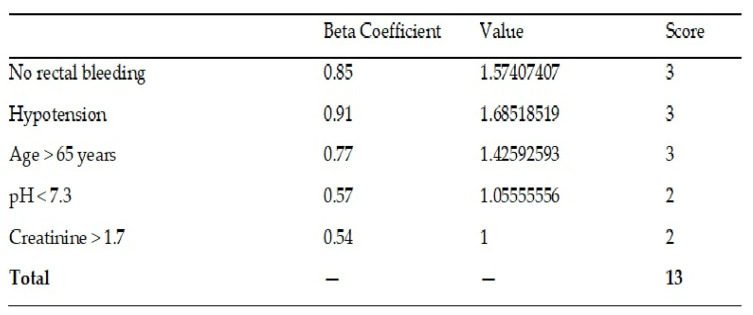
Score obtained from the beta coefficients.

Once the model is obtained by multivariate logistic regression, a score is obtained from the beta coefficients (Figure 2). The predictive capacity of score (including the values of specificity, sensitivity, PPV, NPV) is verified in the derivation subcohort.

**Figure 3:**
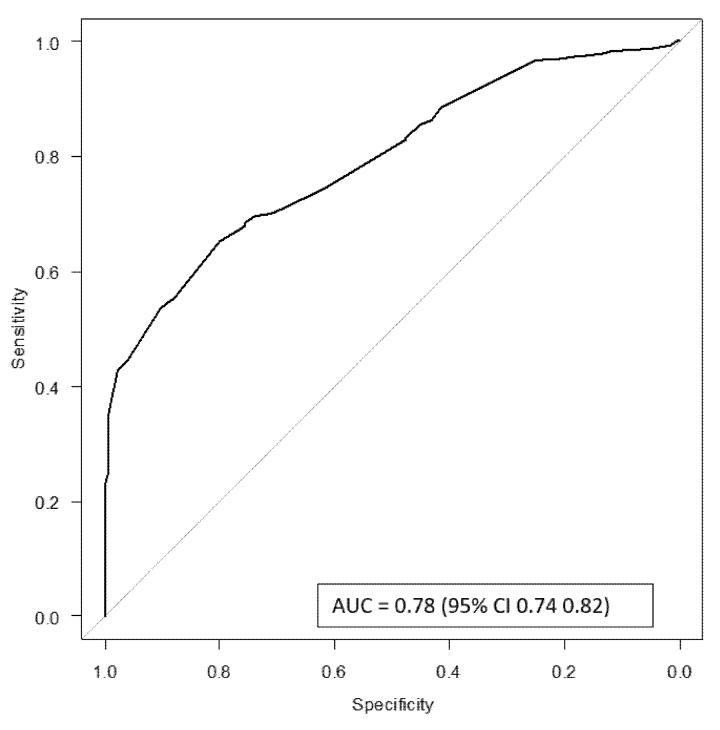
ROC curve.

The area under the curve (AUC) was 0.78, 95% (CI 0.74–0.82), which indicated good discrimination. The Hosmer–Lemeshow test indicated adequate goodness of fit (*p* = 0.391) (Figure 3).

**Figure 4:**
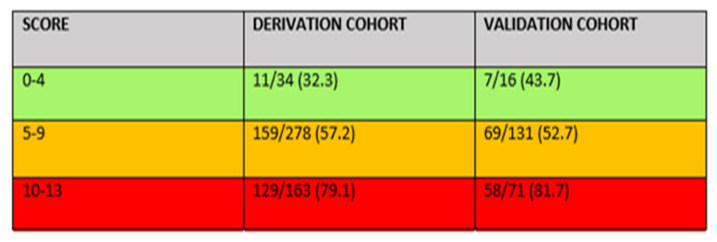
Mortality Prediction Score. Radial Index ≥ 10 Indicating Limitation of Therapeutic Effort.

The score obtained is quite robust and allows us to identify three groups of patients with mortalities between 30 and 40% in the first group, between 50 and 60% in the second group, and 80% in the third group. This could facilitate decisions in the clinical management of these patients. For example, for those patients who are in the third group and have high mortality probabilities, a conservative approach would be the right decision (Figure 4).

## 4. Discussion

Acute mesenteric ischemia (AMI) continues to represent one of the most challenging and lethal abdominal emergencies encountered in clinical practice [14]. Its wide spectrum of clinical presentations, the non-specificity of early symptoms, and the rapid progression to irreversible intestinal damage contribute to its persistently high mortality rates. The present study highlights the remarkable burden of AMI among older adults: more than 70% of the patients in our cohort were aged ≥ 80 years, and over 60% were pluripathological [2,3]. These findings emphasize the increasing relevance of this condition in Internal Medicine departments, where clinicians frequently confront frail, multimorbid patients whose physiological reserve is already significantly reduced.

Given this demographic profile, the decision to pursue surgical or endovascular interventions must be carefully considered [4]. While revascularization and bowel resection remain the cornerstone of curative treatment, invasive management does not uniformly translate into improved survival, particularly in elderly patients with advanced comorbidity and frailty [15]. Instead, aggressive intervention may expose selected patients to substantial perioperative risk, prolonged intensive care, and limited chances of meaningful recovery. This clinical profile closely mirrors that reported in large contemporary series and meta-analyses, in which patients with acute mesenteric ischemia (AMI) are predominantly elderly, carry a substantial burden of cardiovascular risk factors, and experience extremely high in-hospital mortality rates, frequently exceeding 60% and rising above 70% among very elderly individuals. In our cohort, the median age was 81 years, with nearly one-third of patients aged ≥ 85 years—a demographic distribution consistent with prior population-based studies and one that likely contributes significantly to the poor prognosis observed [8].

Cardiovascular comorbidities were highly prevalent in the study cohort, in line with rates reported in previous series. Hypertension affected the vast majority of patients, followed by diabetes mellitus, dyslipidemia, and active or prior smoking, consistent with a typical atherosclerotic risk profile. In addition, nearly three quarters of patients had a documented history of cardiovascular disease, including ischemic heart disease, cerebrovascular disease, and peripheral arterial disease, highlighting the widespread vascular burden associated with acute mesenteric ischemia. Other frequent comorbid conditions, such as chronic kidney disease, heart failure, and atrial fibrillation, all of which are independently linked to poorer outcomes, were common and further increased clinical complexity while narrowing therapeutic options in this high-risk population [8].

Consistent with these baseline characteristics, conservative management predominated both in our cohort and in previously published series, while surgical or endovascular revascularization strategies were largely reserved for younger patients with fewer comorbidities and a more favorable physiological reserve. In our study, fewer than one-third of patients underwent invasive treatment, a proportion similar to that reported in real-world observational studies. This treatment pattern likely reflects delayed diagnosis, advanced disease at presentation, and the high operative risk associated with extensive comorbidity and advanced age [16].

In this context, the accurate identification of patients who are most likely to benefit from invasive treatment and, conversely, those for whom conservative or palliative management may be more appropriate, remains a central unmet need in the clinical care of acute mesenteric ischemia [10].

Although current ACC/AHA guidelines [17] focus primarily on acute limb ischemia rather than acute mesenteric ischemia, they emphasize that acute arterial ischemic syndromes are fundamentally time-dependent conditions associated with high mortality, especially in elderly patients with cardiovascular comorbidities. These recommendations highlight the importance of early risk stratification and prompt, well-informed decision-making. This conceptual framework is directly applicable to AMI, where delays in diagnosis or inappropriate escalation of care can have devastating consequences. Our findings reinforce the notion that early and accurate prognostic assessment is essential to guide timely and proportionate therapeutic strategies in this vulnerable population.

Previous studies have explored the prognostic value of biochemical, clinical, and radiological markers in AMI, yet most have focused on postoperative mortality rather than on guiding the initial decision to operate. For example, recent large retrospective analyses have identified age, ASA score, renal dysfunction, metabolic acidosis, vascular occlusion, and colonic involvement as relevant predictors of adverse outcomes after surgery for AMI. These observations are largely consistent with our findings and support the role of systemic organ dysfunction and disease extent as key determinants of prognosis. However, these studies primarily aimed to predict survival after surgery rather than to assist clinicians in selecting candidates for invasive treatment at the time of presentation [17,18,19].

In contrast to existing prognostic models, our study addresses a more fundamental clinical dilemma: whether invasive management is likely to offer meaningful benefit in the first place [20,21]. By integrating variables from different clinical domains, vital signs, laboratory parameters, and radiological features, the RADIAL score provides a pragmatic and clinically intuitive tool to support early decision-making. Furthermore, most published studies are single-center experiences, limiting the generalizability of their findings [22,23]. By contrast, our multicenter design, encompassing ten hospitals, offers a broader and more representative perspective on the real-world clinical spectrum of AMI. It reflects real-world heterogeneity in patient profiles and management strategies, overcoming a key limitation of many previously published single-center series.

Another relevant aspect of the current study is the integration of geriatric assessment concepts into prognostic reasoning. Although several frailty and comorbidity tools are well established in the care of older adults, none are specific to AMI. In routine practice, clinicians frequently rely on instruments such as the Geriatrics Index of Comorbidity (GIC) [24], which has demonstrated strong predictive value in elderly patients with acute illness by capturing both the presence and severity of comorbidities. Similarly, the Hospital Frailty Risk Score (HFRS) and the Charlson Comorbidity Index (CCI) [25] are widely used to quantify frailty and comorbidity burden and have consistently been linked to worse outcomes in older populations. However, while these tools offer valuable context, they do not address the particular pathophysiological mechanisms and rapid time dependence characteristic of AMI. Their lack of specificity underscores the need for prognostic models tailored to this condition [10].

Beyond comorbidity and frailty indices, several clinical and laboratory parameters have been associated with increased mortality in AMI, including advanced age, hypotension, elevated lactate, leukocytosis, anemia, and renal dysfunction. Nevertheless, many of these markers lack specificity and are influenced by common comorbidities present in elderly patients [22]. Lactate, in particular, is frequently cited as a prognostic marker and is often used to guide the urgency of intervention [26]. However, it reflects global hypoperfusion rather than intestinal ischemia specifically, and in our study, it did not emerge as an independent predictor of poor outcome. Although lactate elevation was associated with mortality in univariate analyses, it did not emerge as a standalone independent predictor in our multivariable models, suggesting that its prognostic value is context-dependent and closely intertwined with other markers of systemic failure.

By contrast, parameters reflecting metabolic and organ dysfunction—such as elevated creatinine and severe acidosis—demonstrated a stronger and more consistent association with mortality. These findings align with prior reports showing that renal impairment and metabolic derangement may be more informative indicators of irreversible disease and poor physiological reserve than lactate alone. In this regard, our results parallel those reported in recent European cohorts, where decreased pH and renal dysfunction were among the strongest predictors of postoperative mortality, reinforcing the central role of systemic organ failure in determining outcomes in AMI [20].

Radiological assessment remains indispensable in the diagnosis and staging of AMI. Computed tomography angiography provides crucial information regarding vascular patency, bowel viability, and secondary signs of advanced ischemia. While pneumatosis intestinalis and portomesenteric venous gas are traditionally regarded as markers of severe disease, their prognostic implications have been inconsistently reported. In our study, radiological findings alone were insufficient to fully stratify risk, supporting the concept that imaging must be interpreted in conjunction with clinical and laboratory data. The strong association between portomesenteric venous gas and mortality observed in our cohort underscores its role as a marker of advanced intestinal injury when present within a broader context of systemic compromise [27].

The RADIAL score developed in this study addresses this gap by combining variables from different clinical domains, vital signs, laboratory parameters, and radiological features, into a unified prognostic score. Our findings demonstrate that higher RADIAL scores correlate with substantially increased mortality risk, providing clinicians with a practical and intuitive tool to guide therapeutic decisions. The model suggests that patients with low scores (0–4) may benefit most from urgent surgical intervention, those with intermediate scores (5–9) require individualized assessment balancing risks and potential benefits, and those with very high scores (10–13) are unlikely to survive invasive management and may be better served with conservative or palliative approaches. This stratified approach aligns with contemporary principles of personalized and value-based care, particularly in elderly and frail populations.

Several limitations should be acknowledged. First, although the overall sample size was large, the number of patients specifically presenting with vascular occlusive acute mesenteric ischemia was relatively small, which may introduce selection bias. In addition, the inclusion criteria did not allow differentiation between embolic and thrombotic etiologies, and detailed radiological severity parameters, such as imaging signs of established intestinal necrosis, were not uniformly available across participating centers, which may limit prognostic granularity. Second, the retrospective design of the study represents an inherent limitation, as it is subject to missing data and unmeasured confounding. In particular, therapeutic decisions regarding conservative versus invasive management may have been influenced by factors not captured in the dataset, including patient frailty, comorbidity burden, functional status, and physician clinical judgment. Consequently, prospective, ideally multicenter, studies incorporating standardized radiological reporting and structured frailty assessments are required to validate and further refine the proposed scoring system and to confirm its clinical applicability in real-world settings.

Despite these limitations, this study has several strengths, including a multicenter design, a large sample size, and the incorporation of clinical, laboratory, and radiological variables into a practical and clinically meaningful prognostic score. These findings provide a valuable contribution to the management of acute mesenteric ischemia and may support clinicians in making complex decisions between invasive and conservative treatment strategies, particularly in elderly and frail patients who are most likely to benefit from accurate and individualized risk assessment.

## 5. Conclusions

The RADIAL score represents a practical, disease-specific tool for acute mesenteric ischemia that facilitates early identification of hospitalized patients with a low likelihood of benefiting from invasive treatment. By integrating readily available clinical, laboratory, and radiological parameters, it may help clinicians avoid potentially futile surgical interventions. Its ease of application across most hospital settings supports faster, more objective decision-making in a population characterized by exceptionally high mortality. Further external validation will be essential to define its role in routine clinical practice.

## Figures and Tables

**Table 1:** Demographic characteristics and personal medical history.

Variable	n (%)
Cardiovascular risk factors	
Hypertension	77.0
Type 2 diabetes mellitus	43.0
Dyslipidemia	41.0
Smoking	30.7
Obesity	21.0
Previous cardiovascular disease (any)	74.1
Ischemic heart disease	28.0
History of stroke	21.0
Peripheral arterial disease	25.1
Other comorbidities	
Chronic kidney disease	33.8
Heart failure	25.8
Atrial fibrillation	28.7
Malignancy	12.9

**Table 2:** Predictive performance of the score. Area under the curve (AUC): 0.78; 95% Confidence Interval: 0.74–0.82—Derivation cohort.

Score	Proportion of Patients	Sensitivity	Specificity	PPV	NPV	Accuracy
≥2	99.6%	99.7%	0.6%	63.0%	50.0%	62.9%
≥3	98.7%	99.0%	1.7%	63.1%	50.6%	62.9%
≥4	93.5%	96.7%	11.9%	65.1%	67.7%	65.3%
≥5	92.8%	96.3%	13.1%	65.3%	67.6%	65.5%
≥6	85.3%	91.3%	25.0%	67.4%	62.9%	66.7%
≥7	69.9%	77.6%	43.2%	69.9%	53.1%	64.8%
≥8	64.8%	72.2%	47.7%	70.1%	50.3%	63.2%
≥9	40.4%	50.2%	76.1%	78.1%	47.3%	59.8%
≥10	34.3%	43.1%	80.7%	79.1%	45.5%	57.1%
≥11	18.7%	24.4%	90.9%	82.0%	41.5%	49.1%
13	8.0%	11.7%	98.3%	92.1%	39.6%	43.8%

**Table 3:** Univariate analysis of pathology and biomarker predictors in relation to death.

Variable	*p* Value
Cardiovascular risk factors (overall)	>0.05
Chronic kidney disease	0.01
Previous stroke	0.013
Hypotension at admission	<0.05
Rectal bleeding at admission	<0.05
Laboratory parameters	
pH < 7.30	0.001
Glucose > 230 mg/dL	0.003
Leukocytosis > 10,000 cells/µL	0.0002
CPK > 400 U/L	0.02
Urea > 43 mg/dL	0.001
Creatinine > 1.7 mg/dL	0.01
CRP > 50 mg/L	0.004
LDH > 875 U/L	0.002
Lactic acid (continuous)	0.24
Lactic acid (categorical)	0.67

## Data Availability

The original contributions presented in this study are included in the article. Further inquiries can be directed to the corresponding author.

## Abbreviations

AMI	Acute mesenteric ischemia
GIC	Geriatrics Index of Comorbidity
HFRS	Hospital Frailty Risk Score
CTA	computed tomography angiography
CCI	Charlson Comorbidity Index
